# Mapping evidence on postpartum modern family planning service uptake among women in Ethiopia: A scoping review

**DOI:** 10.3389/fgwh.2022.1043034

**Published:** 2022-12-23

**Authors:** Niguss Cherie, Mulumebet Abera, Gurmesa Tura

**Affiliations:** ^1^ Reproductive and Family Health Department, School of Public Health, College of Medicine and Health Sciences, Wollo University, Dessie, Ethiopia; ^2^ Population and Family Health Department, Faculty of Public Health, Institute of Health, Jimma University, Jimma, Ethiopia

**Keywords:** postpartum family planning, Ethiopia, scoping review, maternal and child health, contraceptive use, controlled trials or longitudinal studies

## Abstract

**Background:**

In Ethiopia, different fragmented studies have been conducted to assess the determinants and uptake of postpartum modern family planning services. There is discrepancy and inconsistency among reported studies on postpartum modern family planning service uptake. The scoping review aimed to collect evidence on postnatal birth control service use and supply a chance to spot key ideas and gaps to research, policy revision, and changes in strategies.

**Methods:**

There were different process steps in this scoping review which included analysis questions, distinctive relevant studies, study choice, charting the information, and eventually collating, summarizing, and reporting the results. A search was conducted through scientific databases like PubMed, Cumulative Index to Nursing and Allied Health Literature (CINAHL), Hinari, and Google Scholar. The first outcome of this scoping review was postpartum family planning service uptake after childbirth in Ethiopia. The Preferable Reporting Information in Systematic Review and Meta-Analysis (PRISMA) flow diagram was used to select and summarize the selection procedure of the articles. The information of the chosen studies was sorted using the subsequent categories: authors, year of publication, study location, main study objective, and method employed for information analyses.

**Results:**

A total of 1,607 records were reclaimed from the database searches and reference list review. A total of 596 articles were identified in PubMed, 375 in CINAHL, 576 in Hinari, and 60 records in Google Scholar. A total of 1,607 literature studies were checked for replication, and 840 records were excluded. The bulk of articles (*n* = 420) were excluded because they did not focus on postpartum family planning service, and 322 articles were excluded due to study setting discrepancy. The remaining 28 full-text articles were read in full using the preidentified inclusion criteria and included in the scoping review for analysis.

**Conclusion:**

Generally, this scoping review identified different fragmented and inconsistent research findings on the uptake of postpartum modern family planning in Ethiopia. Almost all studies were observational studies that lack interventional study designs to provide evidence-based interventions to improve postpartum family planning uptake. There is a definite need for further interventional and qualitative research to improve early postpartum family planning service uptake that improves maternal and child health.

## Introduction

Postpartum contraception is outlined as women who have ever used any kind of modern birth control technique at intervals for the primary 6 weeks after giving birth (1). Postpartum women have among the highest unmet need for family planning to promote longer birth intervals. Postpartum family planning (PPFP) contributes to the reduction of unplanned and unintended pregnancy and further contributes to the reduction of maternal and newborn deaths (2).

Globally, over 90% of mothers have needs to prevent pregnancy especially the first year after delivery (3, 4). Across the world, efforts to decrease maternal mortality have gained momentum, significantly in light of the sustainable goal agenda that states by 2030 no country ought to have a maternal mortality magnitude relation above 70 per 100,000 live births (5, 6). Conversely, many mothers begin sexual intercourse within the first month after child birth without contraceptive use (5, 6). Actually, postpartum modern family planning service uptake is inconsistent in sub-Saharan countries including Ethiopia (7–10).

Based on to the World Health Organization (WHO), a technical consultation committee for higher maternal and child health outcomes, a need of a minimum of 2 years interval following a childbirth is suggested before getting pregnant once again (2, 4). In Ethiopia, the risk of narrow pregnancy interval among mothers sexually active in 12–23 months of the postnatal period is 72%; however, it decreases to 64% and 70% for mothers in the first 6 months of the postnatal period (8, 10–13).

According to the Ethiopian Demographic and Health Survey (EDHS) 2019, the maternal mortality quantitative relation was 412 per 100,000 live births (14). By employing a contraceptive, mothers will attain their fertility objective by providing for them to avoid their pregnancies and frontier numbers of births (8, 15–19). Ethiopia is one among the developing countries that has achieved a speedy increase in its trendy contraceptive prevalence rate increasing from 10% in 2005 to 60% in 2019 (20). Despite the increase in contraceptive usage, unmet postpartum modern family planning still remains high (3, 12, 20–24). The postpartum period may be a vital time to deal with unmet family planning needs and to cut back the risks associated with closely spaced pregnancies (4). The recommended appropriate stages to use different family planning methods after childbirth can be immediate postnatal (with 48 h), early postnatal (within 42 days), and extended postnatal periods (within 12 months). Totally, different studies show that some mothers became pregnant early within the postnatal period (19), on condition that there is a start of sexual issues, resulting in mothers seeking FP technique (5, 8).

In Ethiopia, a lot of fragmented studies are conducted to assess the uptake and determinants of postpartum modern family planning service uptake. According to these separate studies, postpartum modern family planning service uptake in Ethiopia ranged from 12.5%–80.3% (6, 8, 12, 19, 20, 25–35). From the reports of those studies, there was a variation and inconsistency associated with the uptake of the service throughout the country (8, 13, 27, 29, 36).

This scoping review aimed to explore gaps on postpartum birth prevention service and inform the event of targeted interventions and services to prevent short pregnancy intervals, which is a cause of maternal and child deaths. The objective of this scoping review is to collect evidence on postnatal birth control service use and supply a chance to spot key ideas and gaps to research, policy revision, and changes in strategies. It is important to extract literature studies to analyze the knowledge gaps and unexplored areas of research for potential intervention plans. The findings of this scoping review can even be accustomed to influence scientific inquiry, policy, and follow postpartum family planning. It is also important to identify research gaps and priority areas to researchers, policymakers, and local planners.

## Methods

### Design

We used different steps to conduct this scoping study that has distinctive role in the research question, study choice, charting the information, and eventually organizing, summarizing, and reporting the results of both quantitative and qualitative studies. Elucidating and linking the aim and analysis question; leveling its implementation with extensiveness and exhaustiveness of the scoping process; extracting data; incorporating a numerical outline, and considering the importance of study findings to policy, follow, or research; and incorporating discussion with stakeholders as a needed data conversation were elements of this scoping study methodology.

### Inclusion and exclusion criteria

Studies were enclosed during this scoping review if they met the following inclusion criteria: studies conducted in Ethiopia, empirical and experimental studies, and every study conducted at the community or health facility level; studies reported the end result of interest postpartum family planning service use; articles published in English and published and unpublished articles were enclosed. Articles that were not readily available for complete access or those that proved to be difficult to contact their primary authors were excluded from this review.

### Search

A search of the scientific literature was conducted and four electronic databases using the inclusion and exclusion criteria identified: PubMed, Cumulative Index to Nursing and Allied Health Literature (CINAHL), Hinari, and Google Scholar. Medical Subject Headings (MeSH) thesaurus and keyword terms will be used both in separation and in combination using Boolean operators like “OR” and “AND” to search for eligible articles to be included in the analysis.

The literature databases were searched using a list of keywords and synonyms. The search engine that used the key phrase “postpartum family planning service use” through phrases, synonyms, key words, and proximity searching searched for two or more words in close proximity to one another. As an example, the PubMed database was searched as follows. Search (“postpartum family planning service use” OR “postpartum family planning service utilization” OR “postpartum family planning service uptake” OR “post-natal family planning service use” OR “post-natal family planning service uptake” OR “post-natal family planning service utilization” AND Ethiopia). Advanced search was done by MeSH terms and all fields. The search for published and unpublished studies was conducted from January 1, 2011, to January 1, 2021, and all the articles published from January 1, 2011, to February 1, 2021, were included in this scoping review.

### Outcome of the study

The primary outcome of this scoping review was postpartum family planning service use after childbirth in Ethiopia. In this review, articles that defined postpartum family planning use as women who were using any modern family planning methods to prevent unintended pregnancy after childbirth was included.

### Selection of studies for review

All studies retrieved through search strategy were imported to EndNote X9 to exclude duplicates. After excluding the duplicated articles, title, abstract and full-text reading was used independently to select studies. The Preferable Reporting Information in Systematic Review and Meta-Analysis (PRISMA) flow diagram was applied to summarize and synthesize the selection procedure and process of the articles.

### Charting of key information and summarizing of results

The information of the chosen studies was sorted in steps with the subsequent categories: authors, year of publication, study location, main study objective, and method employed for information analyses. For all quantitative studies, we had a tendency to additionally chart the uptake estimates for postpartum family planning service use. For qualitative studies, we had a tendency to explore the themes most reported by the authors and the way they were found to clarify the use/nonuse of contraceptives in a given context. All extracted data were mapped in information charting forms. Within the method of synthetizing the findings for this scoping review, the investigator repeatedly reviewed the extracted literature studies.

## Results

### Search result

**Figure 1:**
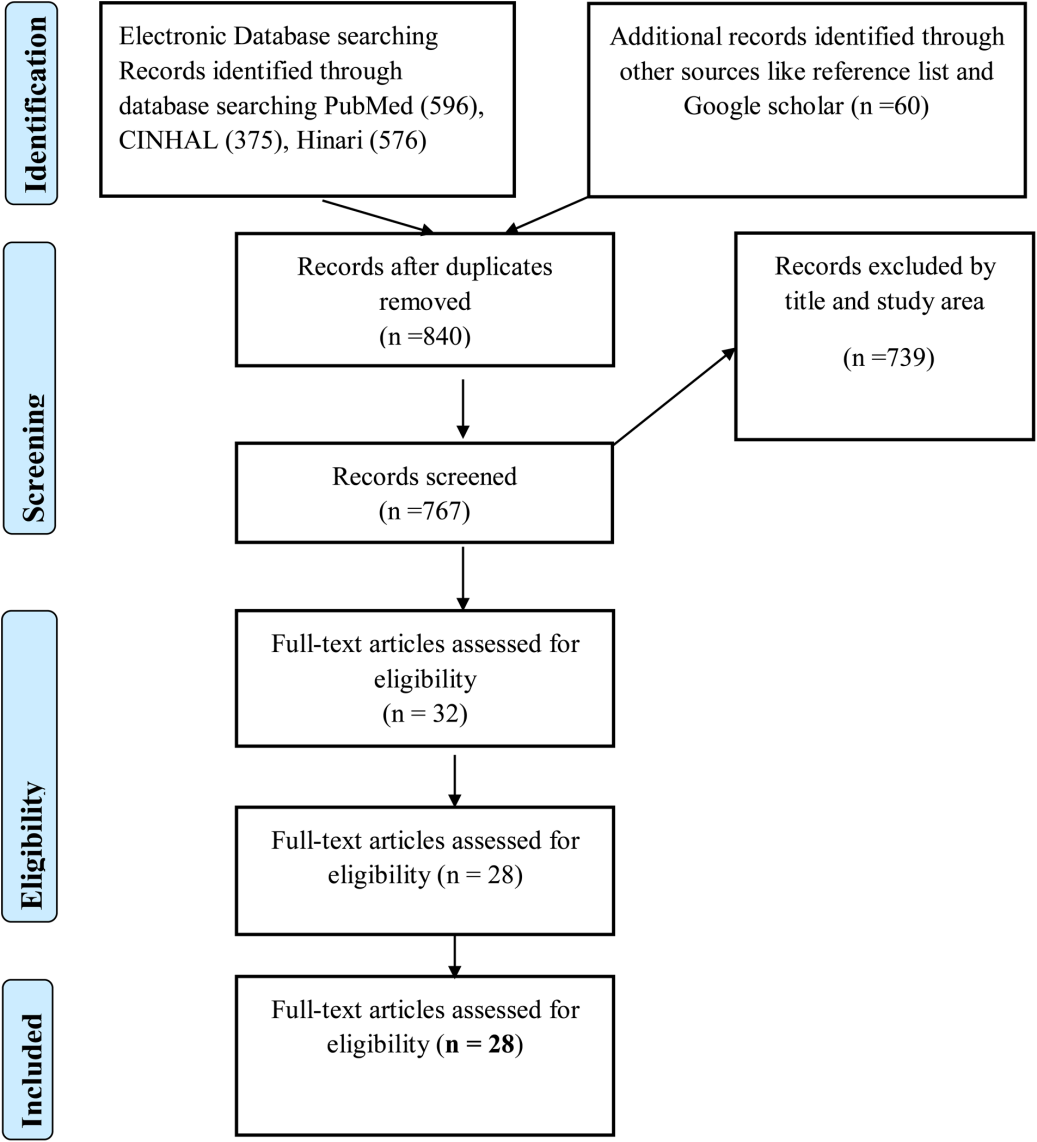
PRISMA flow chart diagram describing the selection of studies for the scoping review of postpartum family planning use among postpartum women in Ethiopia, 2021. PRISMA, Preferable Reporting Information in Systematic Review and Meta-Analysis.

A total of 1,607 records were searched from the catalogue searches and references review. PubMed generated 596 articles, CINAHL generated 375 articles, Hinari generated 576 and Google scholar identified 60 records through reference list review. A total of 1,607 records were assessed for duplication and 840 records were excluded. The bulk of articles (*n* = 420) were excluded because the outcome did not focus on postpartum family planning service use and 319 articles were excluded because the study did not occur in Ethiopia. The remaining 28 full-text articles were read in full using the preidentified inclusion criteria. Based on the inclusion and exclusion criteria, a total of 28 articles were included in this scoping review for analysis. The PRISMA flow diagram summarizes this process (Figure 1).

### Characteristics of original studies

Among the included studies, 21 articles were observational studies with cross-sectional study design, 2 were longitudinal follow-up studies, 2 were quasi-experimental studies, 2 were systematic reviews, and 1 article was a qualitative key informant interview study. A total of 19,322 participants were included in the analysis.

The sample sizes of the investigations range from a minimum of 17 (37) to a maximum of 1,162 (38). Studies in this systematic review reported that the prevalence of postpartum modern family planning utilization among women in Ethiopia ranged from 10.3%, study done at Oromia Regional State (39), to 80.3%, a study done at Addis Ababa (34). Regarding the geographical area, the included 28 studies were done in different regions of the country such as Tigray (4), Amhara (7), Oromia (6), southern nations nationalities and people region (SNNPR) (7), Somali (1), and Addis Ababa city administration (3).

In this scoping review, from the searched and analyzed literature studies, little is known about interventional study designs. There is discrepancy and inconsistency about early postpartum modern family planning service uptake from the literature. Most of the literature studies were centered on extended postnatal birth control services uptake; however, early postpartum modern family planning service uptake with barriers and facilitators was not explored in Ethiopia. No qualitative studies regarded barriers and facilitators of immediate postpartum family planning (IPPFP) service uptake. Access and variations in utilization rates of postnatal birth control of postnatal mothers differed greatly between studies. Objectives, practical implications, and limitations of each included study were identified and reported in the result summary table (Table 1).

**Table 1:** Overview of included studies’ characteristics for postpartum family planning service uptake in Ethiopia: scoping review.

Sr. no	Author, title, year	Setting	Design	Objectives	Sample size	Results	Conclusions	Study limitations
1	Dona et al., Timely initiation of postpartum contraceptive utilization and associated factors among women of child bearing age in Aroressa District, Southern Ethiopia, 2018 (6)	Aroressa District, Southern Ethiopia	Cross-sectional study	Aim of this study was to assess the magnitude and associated factors of timely initiation of postpartum contraceptive utilization among women of child bearing age	695	Timely initiation of postpartum contraceptive utilization was found to be 31.7%. Antenatal care, postnatal care, spousal communication on contraceptive methods, and resumption of menses after delivery were predictors positively associated with timely initiation of postpartum contraceptive utilization	Strengthening integration of family planning information with antenatal and postnatal care follow-up and encouraging spousal communication by promoting information and education	No interventional results to service and system improvement
2	Tg et al., Postpartum family planning utilization among postpartum women in public health institutions of Debre Berhan Town, Ethiopia, 2018 (7)	Debre Berhan Town, Ethiopia	Cross-sectional	To assess family planning use among postpartum women and factors associated with it in public health institutions	248	41.6% women started using contraceptive during postpartum period. Resuming of sex before 6 months and return of menses were significantly associated with utilization of postpartum family planning	Strengthening health education, sexual and family planning counseling, integrating with other service delivery, and promoting PPFP	Small sample size
3	Harrison, and Goldenberg, Immediate postpartum use of long-acting reversible contraceptives in low- and middle-income countries, 2017 (8)	Low- and middle-income countries	Review	To review all literature published from LMIC and summarize the findings	World	In high-, middle-, and low-income countries, there has been an increased interest in the placement of long-acting reversible contraceptives at or immediately after delivery, regardless of delivery mode	Low- and middle-income countries to report on the prevalence of use and satisfaction rates, and note the lack of data on cost and economic implication	Explore data on how future maternal, neonatal, and infant outcomes may be influenced by increased per partum long-term contraceptive use
4	Marta et al. Utilization and associated factors of modern contraceptives during extended postpartum period among women who gave birth in the last 12 months in Gondar town, 2015 (9)	Gondar	Cross-sectional	To assess utilization and associated factors of modern contraceptives during extended postpartum period	404	45.8% mothers used modern contraceptives during postpartum period. Menses returning after birth, resumption of sex, 6–12 months of postpartum period, husband approval of contraceptive, and current on knowledge contraceptive use were associated factors	Strengthening FP counseling service at the ANC clinic and postnatal care would improve contraceptive use during the postpartum period	The study does not include data on the qualitative information of study participants
5	Nigussie, Postpartum family planning utilization and associated factors among women who gave birth in the past 12 months, Kebribeyah Town, Somali Region, Eastern Ethiopia, 2016 (10)	Kebribeyah Town, Somali Region	Cross-sectional	To assess postpartum contraceptive utilization and associated factors in the last 12 months	556	Postpartum contraceptive utilization was 12.3%. Predictors of postpartum family planning utilization were, educational status of respondents, counseling for FP during delivery, duration of postpartum period, and favorable attitude	Health service providers should provide/promote contraceptive service and counseling during PNC, ANC, delivery for postpartum women were recommended	Way of delivery/intervention approach not mentioned
6	Mengesha et al. Contraceptive adoption in the extended postpartum period is low in Northwest Ethiopia, 2015 (13)	Dabat district	Cross-sectional	To determine postpartum contraceptive use and identify the variables which affect postpartum contraceptive use among women	816	A total of 10.3% of the mothers reported adopting contraception in the extended postpartum period. Assistance of a skilled attendant and attended postnatal care services were more likely to use contraceptives	Improving utilization of institutional delivery by a skilled attendant and enhancing postnatal care services are important to increase contraceptive use in the extended postpartum period	Missed confounders in planning the research
7	Taye et al, Prevalence of post partum modern family planning utilization and associated factors among postpartum mothers in Debre Tabor, 2018 (15)	Debre Tabor	Cross-sectional	Assess the prevalence of postpartum modern family planning utilization and associated factors among postpartum mothers	550	Postpartum modern contraceptive was 63%. Age of the mother, married women, return of menses, and history of family planning were the factors positively associated with utilization of PPFP	Need of attention on cause effect relationship studies	Utilization status was determined at a point in time which may slightly lower the prevalence. No conclusion
8	Sitrin et al, Effect of integrating postpartum family planning into the health extension program in Ethiopia on postpartum adoption of modern contraception, 2020 (16)	Oromia Regional State	Quasi-experimental study	Effect of systematically integrating messages on PPFP into community contacts with pregnant and postpartum women	772	Women who delivered at home in the intervention arm were 45% more likely to adopt contraception. There was no difference by arm for women who delivered in a facility	Integrating PPFP into community-level services for pregnant and postpartum women and infants may have additional benefit on top of PPFP services at facilities	Confounders not controlled and no control group and randomization
9	Tegegn et al., Unmet need for modern contraceptives and associated factors among women in the extended postpartum period in Dessie, 2017 (24)	Dessie, Amhara		Objective of this study was to assess unmet need for modern contraceptives and associated factors among women during the extended postpartum period	383	44% of the extended postpartum women had unmet need of modern contraceptives of which 57% unmet need for spacing and 43% for limiting	There is a need to improve the quality of maternal health service, girl's education, and information on postpartum risk of pregnancy to enable mothers make informed choices of contraceptives	Health system and the service providers, the sociocultural factors, and related misconception on family planning did not assess in this study
10	Abera et al, Postpartum contraceptive use in Gondar town, Northwest Ethiopia: a community based cross-sectional study, 2015 (25)	Gondar	Cross-sectional	The aim of assessing the contraceptive behavior of women in the postpartum period	703	48.4% of the postpartum women were contraceptives. The most commonly used method was injection (68.5%). Resumption of menses, age ≤24 years, duration of 7–9 months after delivery, and having antenatal care were the factors positively associated PPFP	Strengthening family planning counseling during antenatal care visit and postnatal care would improve contraceptive use in the postpartum period	The sociocultural factors and related misconception on family planning did not assess in this study
11	Gonie et al, Acceptability and factors associated with post-partum IUCD use among women who gave birth at bale zone health facilities, 2017 (26)	Bale, Oromia	Cross-sectional	Determine the level of acceptability and factors associated with immediate PPIUCD use among women who gave birth	465	The acceptance of immediate PPIUCD usage was 12.4%. The odds of accepting PPIUCD insertion was higher among women who attended three antenatal care visits than those who did not attend antenatal care visits for the current birth	Due attention should be given to enhancing the educational level of women and effective IUCD counseling should be given during antenatal care visits to correct misconceptions and fears of complication about PPIUCD insertion	Did not address cultural barriers
12	Mihretie et al, Postpartum modern contraceptive utilization and associated factors among women who gave birth in the last 12 months in Addis Zemen, South Gondar, Ethiopia: community-based cross-sectional study, 2020 (27)	Addis Zemen, Gondar	Cross-sectional study	To assess postpartum modern contraceptive utilization and associated factors among postpartum women	402	The prevalence of postpartum family planning utilization was 54.7%. Maternal educational status, menses return, less than four alive children, postnatal care follow-up, length of time after delivery, and knowledge were significantly associated with postpartum modern contraceptive utilization	Healthcare providers should strengthen the integration of family planning services with maternal and child health service, provide health information about timely use of contraceptives, and improve postnatal care follow-up after giving birth	Memory bias might be introduced and this study's focus on quantitative approach which could not address the “why” questions in detail
13	Abebe and Mannekulih, Focused family planning counseling increases immediate postpartum intrauterine contraceptive device uptake, 2020 (28)	Adama, Oromia	Quasi-experimental study	Assess the effectiveness of FFPC in increasing PPIUCD uptake among mothers	726	The proportion of PPIUCD uptake in the IG 12.4% and NIG 4.8%. Being unmarried women, making a mutual decision, and having a better knowledge of family planning were factors associated with uptake	The currently implemented counseling approaches should be revised so that they can be efficiently integrated and implemented in the existing context of the health service delivery	Social desirability bias
14	Abraha et al., Predictors of postpartum contraceptive use in rural Tigray region, northern Ethiopia: a multilevel analysis, 2015 (30)	Aksum, Tigray	Cross-sectional	Aimed to assess postpartum modern contraceptive use and associated factors among postpartum women	601	48% of women used modern contraceptive during extended postpartum period. Family planning counseling during prenatal and postnatal care, having postnatal care, resuming sexual activities, and menses returned after birth. Experiencing problem with previous contraceptive use was negatively associated with modern contraceptive use	Strengthening family planning counseling during prenatal care and postnatal care visits, improved postnatal care services utilization	No interventional recommendations
15	Woldu et al., Long-acting reversible contraception utilization and associated factors among women in extended postpartum period in Hossana town, southern Ethiopia: cross sectional study (31)	Hossana town, southern Ethiopia	Cross-sectional study	Aimed at assessing the prevalence and factors associated with LARC use among postpartum women	381	The prevalence of LARC use was 36.5%. The main reason for not using LARC was fear of side effects and false information. Previous use of LARC and discussion with health providers on LARC were found to be significantly associated with LARC use	Provision of effective contraceptive counseling during antenatal, delivery, and postnatal care services with emphasis on LARC/postpartum intrauterine device is important	The study hinders in-depth exploration of women's perception and barriers for LARC use. Finding might not be representative for the rural communities
16	Gebeyehu et al., Postpartum modern contraceptive use and associated factors in Hossana town, 2019 (32)	Hossana town, SNNPR	Cross-sectional	The main purpose of this study was to determine postpartum modern contraceptive use and associated factors in Hossana	368	72.9% women used postpartum modern contraception. Educational status of mother, resumption of sex, menses resumption, and duration postpartum period had significant association with postpartum modern contraceptive use	Improving women education and delivering messages for couples on the risk of getting pregnant prior to menses is crucial	The limitation of this study includes better to incorporate institutions delivering service, healthcare providers, and male partners
17	Gebremedhin et al., Family planning use and its associated factors among women in the extended postpartum period in Addis Ababa, Ethiopia, 2018 (34)	Addis Ababa	Cross-sectional	To assess postpartum family planning use and its associated factors among women in extended postpartum period	803	The prevalence of postpartum family planning use was 80.3%. Marriage, menses resumption after birth, length of time after delivery, and history of contraceptive use before last pregnancy, were the factors associated PPFP use	Special emphasis to those who had no history of contraceptive use and exposure to the other identified factors	Factors relating to the health system and service providers not addressed
18	Abraha et al., Intentions on contraception use and its associated factors among postpartum women in Aksum town, Tigray region, northern Ethiopia: a community-based cross-sectional study(35)	Aksum town, Tigray region	Cross-sectional study	Assess intention to use modern contraceptive and to identify factors associated among postpartum women	604	Intention to use modern contraceptive was 84.3%. Resumed sexual intercourse and women whose husband approved family planning to use and knowledge were more likely to have intention on contraceptive use	Family planning services providers and programmers should continue the promotion of partner involvement and Increasing family planning knowledge through printed media and mass media	Difficult to identify cause effect relationships
19	Ababa et al., Factors affecting long-term and permanent contraceptive uptake among immediate post-partum mothers at Saint Paul’s Hospital Millennium Medical, 2018 (36)	Addis Ababa	Cross-sectional	Barriers to uptake of long-term and permanent family planning methods among immediate postpartum mothers	422	45% of the women accepted long-term and permanent contraception on their immediate postpartum period before discharge	Postpartum programs need to strengthen such service	Facility factors not addressed
20	Asnake et al, Utilization of maternity waiting homes to increase uptake of immediate postpartum family planning in primary health care facilities in Ethiopia, 2017 (40)	Amhara, Oromia, SNNPR and Tigray	Cross-sectional	To assess the contribution of MWHs to increase IPPFP uptake among women who deliver in health facilities in Ethiopia	884	IPPFP use among women who used MWHs was 44% and 36% among those who did not use MWHs. The use of MWHs significantly contributed to increase immediate postpartum family planning uptake	MWHs contributed for improving IPPFP uptake by an average of 8%-point difference for beneficiaries within 48 h after delivery	This study is limited by its focus on USAID transform agenda
21	Mehare et al., Postpartum contraceptive use and its determinants in Ethiopia: a systematic review and meta-analysis (41)	Ethiopia	SRMA	To estimate the pooled prevalence of postpartum contraceptive use and determinants in Ethiopia using the accessible studies	18 studies	The pooled prevalence of family planning use among mothers during the postpartum period in Ethiopia was 48.11%	The health sector policymaker, promoters, and providers should give attention to mothers	The confounding variables most of the time might affect the outcome variable
22	Tafere et al, Counseling on family planning during ANC service increases the likelihood of postpartum family planning use in Bahir Dar City Administration, Northwest Ethiopia, 2018 (42)	Bahirdar	Prospective follow-up study	Investigating the role of family planning counseling during antenatal care in promoting postpartum modern family planning use within 6 weeks after birth	823	Postpartum modern family planning use within 6 weeks after delivery among the study women was 19%. Among mothers counseled, 38.5% of them used postpartum modern family planning compared to 13.4% of postpartum women who were not counseled	Health providers need to ensure continuity of care through strengthening integration of family planning counseling services during ANC and referral linkages between community and health workers	This study might also have selection bias and quality of counseling not measured
23	Zimmerman et al, Effect of integrating maternal health services and family planning services on postpartum family planning behavior in Ethiopia: results from a longitudinal survey, 2019 (43)	SNNPR	Longitudinal survey	To establish whether PPFP counseling is being provided in antenatal and postnatal care services and whether receipt of PPFP counseling improved uptake of postpartum family planning use by 6 months postpartum	329	By 6 weeks postpartum, only 20% of women had received counseling. There was no difference between women who received PPFP counseling only in ANC and women who did not receive counseling at all	Integration of postpartum family planning counseling into postnatal care services is an effective means to increase postpartum contraceptive uptake	Limited confounding due to temporal changes that are present when including all births over a 3- or 5-year period, which may mask more recent progress
24	Teka et al., Role of antenatal and postnatal care in contraceptive use during postpartum period in western Ethiopia: a cross sectional study, 2018 (44)	Gida Ayana district, Oromia regional state	Cross-sectional study	To assess the magnitude and determinants of contraception utilization in extended postpartum period	603	Proportion of women using any of the modern family planning in extended postpartum period was 45.4%. Women who had four and more antenatal care visits, mothers who received postnatal care, and those desiring less number of children were more likely to use modern family planning methods during the extended postpartum period	Healthcare providers should work to improve quality of health services provided during antenatal care and postnatal care to enhance family planning utilization among postpartum women	Recall bias might have been introduced and Social desirability bias
25	Gebremariam and Gebremariam, Contraceptive use among lactating women in Ganta-Afeshum District, Eastern Tigray, Northern Ethiopia, , 2015: a cross sectional study, 2015 (45)	Tigray	Cross-sectional study	To determine the magnitude of modern contraceptive utilization and factors associated with it among lactating women	605	The magnitude of MC utilization was 68.1%. The contraceptive method mix was dominated by Depo-Provera (58.8%) followed by implants (31.8%)	Family planning information dissemination using radio in rural settings should be encouraged to increase the uptake of contraceptives in lactating women	The study did not address all health system related factors and history of prior use of contraceptives
26	Sonalkar et al., Programmatic aspects of postpartum family planning in developing countries: a qualitative analysis of key informant interviews in Kenya and Ethiopia, 2013 (39)	Ethiopia	Key informant interview	To determine the perceptions regarding programmatic aspects of postpartum family planning by key informants in 17 countries determined to have high unmet need for postpartum family planning	17	In Ethiopia, all interviewees described the challenge of delivering postpartum family planning services to the 90% of women who deliver away from a health facility	Support for improved tracking systems of contraceptive method use in health centers will improve the ability to accurately supply commodities. In addition, additional research will allow improved advocacy for postpartum family planning	A limitation of this study is its small sample size
27	Jima and Garbaba. Postpartum Family Planning Utilization and Associated Factors Among Women Who Gave Birth in the Last 12 Months Prior to the Study in Lode Hetosa District, South East Ethiopia, 2018 (37)	Lode Hetosa District, South East Ethiopia	Cross-sectional	To assess magnitude of postpartum family planning utilization and its associated factors among postpartum women who gave birth within 1 year prior to the study	1,162	Magnitude of postpartum family planning utilization in the study area was 15%. The most frequently used family planning method within 6 weeks after delivery was implants (35%)	The findings of this study can imply that, healthcare workers have to provide counseling services to pregnant women so that they can uptake family planning method immediately after delivery	Perspective of men was not considered except in qualitative part. This could have recall biases
28	Tessema et al, Association between skilled maternal healthcare and postpartum contraceptive use in Ethiopia, 2016 (38)	Ethiopia, EDHS,	Cross-sectional	Aims to assess the association between skilled maternal healthcare and postpartum contraceptive use in Ethiopia	5,000	In 2016, postpartum contraceptive use was significantly associated with an institutional delivery and skilled antenatal care. No significant relationship was observed in either survey round between postpartum contraceptive use and skilled delivery or postnatal care	National efforts to strengthen the integration of family planning counseling and services with skilled maternal healthcare services are succeeding	No qualitative evidence of cultural barriers

EDHS, Ethiopian Demographic and Health Survey; PPFP, postpartum family planning; IPPFP, immediate postpartum family planning; IG, intervention group; NIG, nonintervention group; PPIUCD, postpartum intrauterine contraceptive device; IUCD, intrauterine contraceptive device; FFPC, focused family planning counseling; MC, modern contraceptives; LMIC, low and middle income countries; FP, family planning; ANC, ante natal care; PNC, post natal care; LARC, long acting reversible contraceptives; MWH, maternal waiting home; USAID, United States action aid.

### Gaps in literature studies

**Figure 2:**
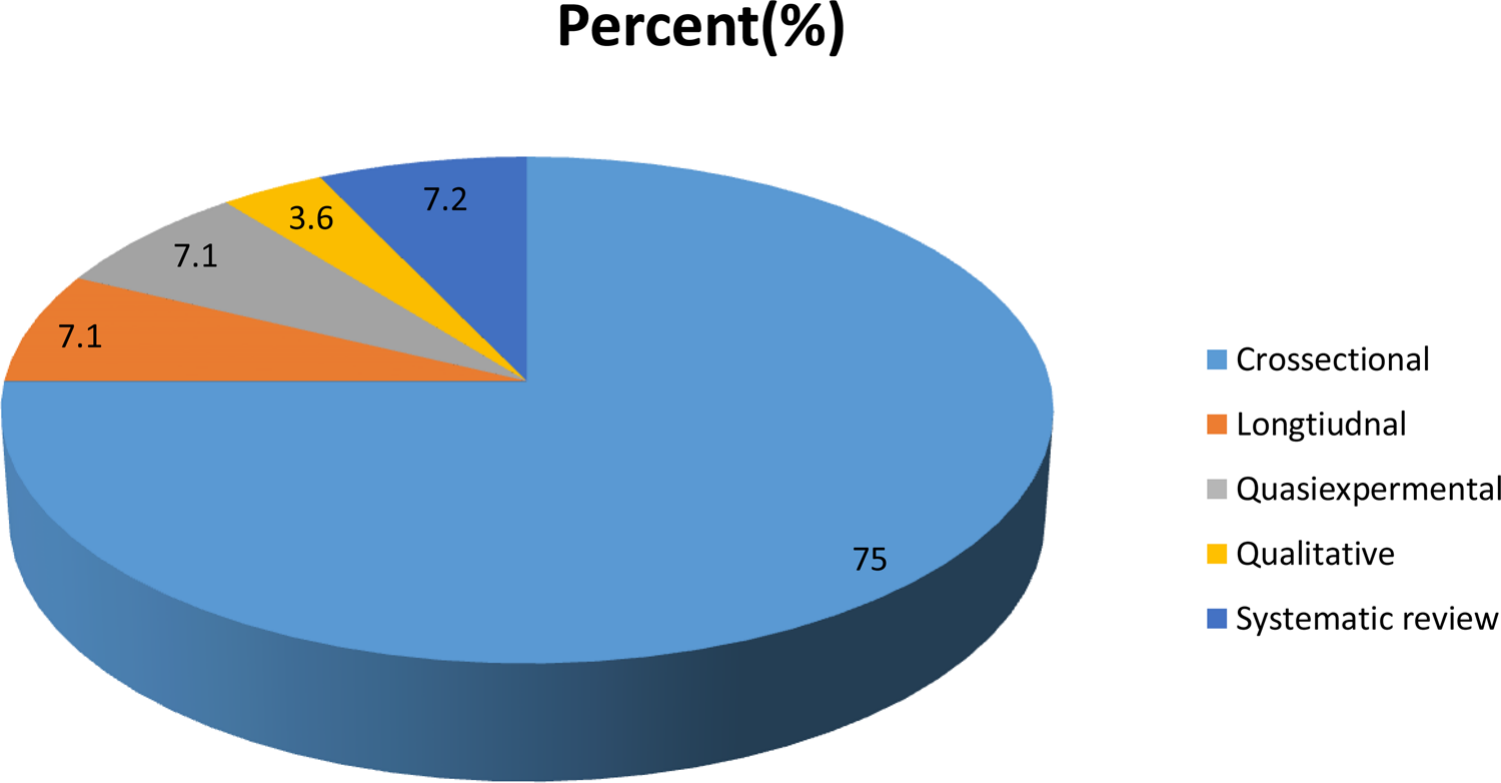
Summarized study designs in the scoping review on mapping evidence on postpartum modern family planning service uptake among postpartum women in Ethiopia, 2021.

In this scoping review, from the searched and analyzed literature studies, little is known about interventional study designs (Figure 2). There is discrepancy and inconsistency about early postpartum modern family planning service uptake from the literature. Most of the literature studies were centered on extended postnatal birth control services uptake; however, early postpartum modern family planning service uptake with barriers and facilitators was not explored in Ethiopia. There was a lack of qualitative studies concerning barriers and facilitators of early postpartum family planning service uptake.

## Discussion

A wide knowledge and research gap on postpartum family planning service was identified through this scoping review of the scientific literature. Ethiopia is one of the geographical countries that has achieved a speedy increase in its trendy contraceptive prevalence rate increasing from 10% in 2005 to 60% in 2019 (20). Despite the speedy increase in contraceptive uptake, the unmet need for postpartum birth prevention still remains high (3, 12, 20–24). The postpartum period may be a vital time to deal with unmet family planning needs and to cut back the risks associated with closely spaced pregnancies(4). Totally different studies show that some mothers became pregnant early within the postnatal period (19).

In Ethiopia, a lot of fragmented studies are conducted to assess the uptake and determinants of postpartum contraceptive use. These separate studies showed that uptake of postnatal contraceptive use in Ethiopia ranged from 12.5% to 80.3% (6, 8, 12, 19, 20, 25–35). From the reports of those studies, there was a variation and inconsistency associated with the uptake of postnatal contraceptive use throughout the country (8, 13, 27, 29, 36).

Research articles focus on describing postnatal family planning service use in nonspecific ways in which the bulk of findings on postnatal family planning has targeted on extended postnatal period. There is very little evidence that shows the effectiveness of mobile health interventions to boost immediate postnatal family planning service within the initial 45 days after childbirth that is vital to prevent unwanted pregnancy, unsafe abortions, and planning desires among mothers in Ethiopia.

This scoping review shows the requirement for research inquiry to know the effectiveness of intervention on controlled community trial studies, particularly the application of telemedicine, to boost postnatal family planning service uptake to improve maternal and child health in Ethiopia. A deep investigation of practicability and effectiveness of mobile health interventions for postpartum family planning uptake, avoid barriers, and improve community support is required to advocate postpartum family planning in Ethiopia.

### Limitations of the review

The limitation of the studies was the lack of appropriate study designs/interventional and qualitative studies to provide evidence-based information for improved postpartum modern family planning services uptake and reduce the burden of adverse maternal and child outcomes associated with poor uptake.

## Conclusion

Generally, this scoping review identified different fragmented and inconsistent research findings on the uptake of postpartum modern family planning in Ethiopia. Almost all were observational studies that lacked interventional study designs to provide evidence-based interventions to improve postpartum family planning uptake at early stage of postnatal period to prevent unwanted and narrow birth interval pregnancies.

## Recommendations

This scoping review highlights a definite need for the development of further research to improve immediate postpartum family planning service use that improves maternal and child health. Beyond the existing maternal and child healthcare approach, there is a need for investigation on the effectiveness of mobile health interventions needed to improve immediate postpartum family planning service use. As we continue with our efforts to accelerate the average annual reduction rate in maternal and neonatal mortality and to achieve sustainable development targets, an increased focus on studies of immediate postpartum family planning service use merits immediate attention.
